# Identification and validation of PANoptosis-related LncRNAs prognosis system in hepatocellular carcinoma

**DOI:** 10.1038/s41598-025-90498-y

**Published:** 2025-02-19

**Authors:** Qi Shu, Junfeng Zhu, Jiaping Mo, Xiaoyan Wei, Zhenjie Zhu, Xiaojuan Chen, Fugen He, Like Zhong

**Affiliations:** Zhejiang Cancer Hospital, Hangzhou Institute of Medicine (HIM), Chinese Academy of Sciences, Hangzhou, 310022 Zhejiang China

**Keywords:** Hepatocellular carcinoma, PANoptosis, lncRNA, Immune infiltration, Risk stratification, Prognosis, Cancer, Genetics, Bioinformatics

## Abstract

Hepatocellular carcinoma (HCC) is one of the most common solid malignancies in the world. Due to the limited effectiveness of current drug treatments, further research on HCC is necessary. PANoptosis is defined as an inflammatory RCD whose main features combine pyroptosis, apoptosis and necroptosis which cannot be explained by any of these three RCDs alone. In HCC, risk stratification based on PANoptosis-associated lncRNAs has clinical application potential. In this study, we explored HCC related PANoptosis-related lncRNAs (PRLs) by analyzing significantly differentially expressed genes in HCC. HCC-associated PRL scores were established by WGCNA, LASSO analysis and multivariate Cox assessment. Subsequently, we verified the prognostic analysis ability of PRL score for HCC patients, and on this basis established a prognostic risk assessment model for HCC and verified its reliability. The relationship between PRL score and immune infiltration as well as drug sensitivity was further analyzed to evaluate the clinical reference value of this model. Western blot analysis and PCR further verified the reliability of bioinformatics results. The observed suppression of HCC progression and invasiveness following selected PRL knockdown further validated the reliability of our bioinformatics analysis results. Our results provide new evidence for the role of PANoptosis-associated lncRNAs in HCC.

## Introduction

Hepatocellular carcinoma (HCC) is one of the most common solid tumor malignancies in the world, accounting for approximately 90% of primary liver malignancies^1^. The two most common risk factors for HCC globally are chronic viral infection and alcohol intake^2,3^. Most HCC is diagnosed at an advanced stage and surgery is often limited due to the multifocal nature of the disease and the combination of cirrhosis^4,5^. Due to the limited efficacy of current drug therapy, further research on HCC is necessary^6^.

HCC risk stratification is difficult due to genetic diversity and molecular heterogeneity^6^. The application of new drugs such as tyrosine kinase inhibitors (TKI) and immune checkpoint inhibitors (ICI) is limited by different response levels in different patients^6^. Although there have been reports pointing to the potential of novel prognostic markers such as Wnt, beta-catenin mutations, and Vascular Endothelial Growth Factor A (VEGFA). However, to date, other than Alpha-fetoprotein (AFP), there are no approved biomarkers for predicting response/resistance to systemic therapy with ICI and TKIs^7^. In order to better assess HCC risk and predict efficacy, long non-coding RNA (lncRNA) has received increasing attention^8,9^. By modifying the activity and expression levels of tumor suppressor factors and oncogenes, lncRNAs can be used as biomarkers for HCC efficacy^10^. Therefore, HCC risk stratification based on lncRNA has clinical application potential.

Pyroptosis, apoptosis, and necroptosis are three types of programmed cell death (RCD) that have historically been considered independent. But now there is growing evidence that these RCDs have widespread crosstalk at multiple levels^11^. PANoptosis is now defined as an inflammatory RCD with key features of pyroptosis, apoptosis, and necroptosis that cannot be accounted for by any of these three RCDs alone^12^. PANoptosome was identified as a key protein for induction and regulation of PANoptosis and provided a molecular scaffold required for the process^13^. The key molecules in the formation of PANoptosome and the subsequent PANoptosis process include ZBP1, RIP1, CASP8, NLRP3 and inflammasome^14^. In view of the important impact of PANoptosis on immune response, chemotherapy and PD-1/PD-L1 related treatment, it is of great clinical significance to further study PANoptosis^12,15–17^. At present, no in-depth mechanism of PANoptosis has been reported in HCC.

In this study, we explored the HCC related PANoptosis-related lncRNAs (PRLs) by analyzing the significantly differentially expressed genes in HCC. HCC associated PRL scores were established by WGCNA, LASSO analysis and multivariate Cox assessment. Subsequently, we verified the prognostic value of PRL scores for HCC patients, and on this basis established a risk assessment model for HCC prognosis and verified its reliability. The relationship between PRL score and immune infiltration and drug sensitivity was also analyzed. The observed suppression of HCC progression and invasiveness following selected PRL knockdown further validated the reliability of our bioinformatics analysis results. Our results provide new evidence for the role of PANoptosis-related lncRNAs in HCC.

## Materials and methods

### Data collection and preprocessing

The RNA-Sep data (FPKM, TCGA-LIHC) and corresponding clinical information for HCC samples were obtained from the publicly available The Cancer Genome Atlas (TCGA) database. Under the Perl language environment, the transcriptome data of 424 HCC samples (normal: 50 samples, HCC: 374 samples) were extracted and merged into a file. Combined with clinical information, the HCC samples without survival time were deleted and 370 HCC samples were enrolled for the final analysis. In addition, based on the ICGC database (International Cancer Genome Consortium), we extracted 231 HCC samples with clinical prognostic characteristics to serve as an independent external validation cohort. The mutation files (MAF format) of HCC samples were acquired from the TCGA database via Perl environment.

### WGCNA construction to select the pivotal PRLs for HCC

After the annotation of RNA-Sep data based on the human genome, the expression profiles of mRNA and lncRNA were acquired, respectively. The PANoptosis-related genes (PRGs) were collected from the previous literature and 14 PRGs were identified in this study (Supplementary Table 1). With the cutoff of |coefficient|> 0.3 and *p* < 0.05, 547 lncRNAs were defined as PANoptosis-related lncRNAs (PRLs) (Supplementary Table 2). “igraph” script was carried out to exhibited the relationship of PRGs and PRLs in R environment. Based on “WGCNA” script, the weighted gene co-expression network analysis (WGCNA) was used to explore the pivotal module for HCC from the PRLs. On the basis of expression profile of normal and HCC samples, the data was cleaned to delete the missing value. Then, all the samples were clustered to exclude the outlier samples. Under the threshold of mean FPKM = 0.5, the gene modules were clustered and dynamic merged by dynamic tree cut. Based on the Pearson correlation analysis, the relationship of gene module and clinical traits was explored and the most correlated gene module was chosen for the further analysis.

### PRL score estimation and independent prognosis analysis

105 HCC-associated PRLs were identified by WGCNA and enrolled to calculate the PRL score for HCC (Supplementary Table 3). Univariate Cox analysis was employed to explore the prognostic PRLs for HCC with *p* < 0.05 via “survival” script. “glmnet” script was used to construct the LASSO model to select the feature variables from the prognostic PRLs. Then, on the basis of multivariate Cox analysis, those feature variables were included to calculate the PRL score. The PRL score was estimated as following formula: PRL score = Coefficient x AL442125.2 expression + Coefficient x MIR4435-2HG expression + Coefficient x AC026412.3 expression + Coefficient x LINC01224 expression + Coefficient x AC026356.1 expression. Combined with PRL score and clinical variables, the independent prognosis analysis was investigated using univariate and multivariate Cox analysis via “survival” script. ROC analysis was applied to evaluate the diagnostic ability of PRL score and clinical variables.

### Verification of PRL score-based risk model in training and test groups

“caret” script was carried out to divide the HCC samples into training and test groups with the classification set at 7: 3 and the PRL score of each HCC samples was estimated.

Based on the optimal cutoff value for clinical prognosis in HCC samples, we randomly categorized samples in both the training and validation sets into low and high PRL score subgroups. Additionally, using the optimal cutoff from the clinical survival curves of HCC samples, we classified HCC samples from the independent external ICGC cohort into low and high PRL score subgroups to validate the accuracy and stability of the PRL score prognostic model. “pheatmap” and “survminer” scripts were adopted to explore the association of PRL score and clinical prognosis for HCC. “survivalROC” was performed to estimate the AUC of 1-, 3- and 5 years AUC for HCC. Based on the clinical variables, the HCC samples were divided into different clinical subgroups, and the prognosis of HCC in clinical subgroups with low- and high PRL score was evaluated via script “survival”.

### Development of nomogram based on PRL score and different clinical characteristics

In the two independent PRL score groups, a nomogram was developed to estimate the clinical outcome in 1-, 3-, and 5 years of HCC based on the PRL score and clinical variables via “rms” script. “regplot” script was employed to evaluate the consistency of nomogram predicted prognosis and actual prognosis and visualized in calibration curve. Concordance index (C-index) was used to explore the precision of PRL score and clinical variables in evaluating prognosis for HCC via “pec” and “rms” script.

### Immune infiltration assessment and function enrichment analysis

Single sample gene set enrichment analysis (ssGSEA) was carried out to estimate the immune infiltration landscape of HCC via “GSVA” script. Based on the marker gene of immune cells, the fraction of 23 type immune cells were estimated. Spearman rank correlation analysis was adopted to explore the relationship of PRL score and immune infiltration. With the cutoff set at |fold change|> 2 and p.adjust (FDR) < 0.05, the differential expression genes (DEGs) between PRL score subgroups was estimated. “clusterProfiler” script was carried out to enrich the DEGs into gene oncology (GO) terms. “GSVA” script was employed to calculate the kyoto encyclopedia of genes and genomes (KEGG) terms of each HCC samples in PRL score subgroups, with the threshold set at *p.adjust* < 0.05^18–20^.

### Analysis of mutation landscape and chemotherapy drug prediction

Under the Perl language environment, the MAF files of low- and high PRL score groups were extracted. “maftools” script was employed to explore the mutation landscape of HCC samples in the PRL score subgroups. Based on the genomics of drug sensitivity in cancer (GDSC) database, the response of chemotherapy drug (IC50) was predicted via “pRRophetic” script.

### Cell culture

MIHA cells and Huh7 cells were obtained from the American Type Culture Collection (ATCC). MIHA cells were cultured in Dulbecco’s Modified Eagle Medium (DMEM) supplemented with 10% fetal bovine serum (FBS). Huh7 cells were cultured in DMEM supplemented with 10% FBS and 1% penicillin–streptomycin. Cells were maintained at 37 °C in a humidified incubator with 5% CO₂. The growth of the cells was regularly monitored. When the cells reached 80–90% confluence, they were passaged at a 1:3 ratio using 0.25% trypsin.

### Real-time quantitative PCR (RT-qPCR) analysis

Total RNA was extracted from cells using TRIzol reagent (Thermo Fisher Scientific) according to the manufacturer’s instructions. The quality and concentration of RNA were measured using a NanoDrop 2000 spectrophotometer (Thermo Fisher Scientific). cDNA was synthesized from 1 µg of RNA template using the PrimeScript RT Reagent Kit (TaKaRa Bio). RT-qPCR was performed using SYBR Green PCR Master Mix (Thermo Fisher Scientific) and relative gene expression levels were calculated using the2^^-ΔΔCt^ method with GAPDH as the internal control gene.

### Cell viability assay using MTT

After terminating digestion, centrifuge the cell suspension for 5 min and discard the supernatant. Resuspend the cells in 3 mL of fresh culture medium. Following trypsin digestion and centrifugation, remove the supernatant and resuspend the cells again. Seed the cells at a density of 1 × 10^6 cells per well in a 6-well plate and allow them to adhere for 12 h. After adherence, introduce AC026412.3 interference and culture the cells to the designated time points (0, 24, 48, 72, and 96 h). At each time point, add 20 µL of MTT solution at a concentration of 5 mg/mL to each well, incubate for 4 h, then discard the solution. Subsequently, add 150 µL of DMSO to each well and agitate to ensure thorough dissolution. Measure the absorbance at 570 nm using a microplate reader and plot a growth curve based on the absorbance values at different time points.

### Colony formation assay

Seed the collected cells in culture dishes at a density of 1 × 10^3 cells/mL, mix thoroughly, and place in an incubator for culturing. After 5–7 days, observe under a microscope until each colony contains approximately 10–15 cells. Then, remove the culture dish, wash with PBS, fix with methanol for 30 min, and stain with crystal violet for 2 h. Photograph and count the number of cell colonies.

### Western blot analysis

Total protein was extracted from AC026412.3-interfered Huh7 cells and control group cells using RIPA lysis buffer containing protease and phosphatase inhibitors (Beyotime, China). Protein concentrations were measured with a BCA Protein Assay Kit (Thermo Fisher Scientific, USA). Equal amounts of protein (30 µg per sample) were separated on 10% SDS-PAGE gels and subsequently transferred onto PVDF membranes (Millipore, USA). The membranes were blocked with 5% non-fat milk in TBST buffer (Tris-buffered saline containing 0.1% Tween-20) for 1 h at room temperature, followed by overnight incubation at 4 °C with the following primary antibodies: anti-β-actin (1:1000, #4967, Cell Signaling Technology, USA), anti-Caspase-3 (1:1000, #9662, Cell Signaling Technology, USA), anti-Bax (1:1000, #2772, Cell Signaling Technology, USA), anti-NLRP3 (1:1000, #15101, Cell Signaling Technology, USA), anti-MLKL (1:1000, #14993, Cell Signaling Technology, USA) and anti-p-MLKL (1:1000, #91689, Cell Signaling Technology, USA). After washing the membranes three times with TBST, they were incubated with HRP-conjugated secondary antibodies (1:5000, Beyotime, China) for 1 h at room temperature. Protein bands were visualized using enhanced chemiluminescence (ECL) reagents (Thermo Fisher Scientific, USA) and imaged with a ChemiDoc Imaging System (Bio-Rad, USA). The relative expression levels of the target proteins were quantified using ImageJ software, normalized to β-actin as the internal control.

### Transwell assays

Before the experiment, coat the bottom of the Transwell insert membrane with Matrigel and store at 4 °C for future use. Collect transfected cells, suspend them in serum-free DMEM medium, count, and adjust to an appropriate density, then add them to the upper chamber of the Transwell. Add serum-free medium to the upper chamber and complete medium with serum to the lower chamber, followed by incubation for 48 h. After incubation, remove the Transwell inserts, fix the cells with methanol, and stain with crystal violet. Using an inverted microscope, observe and count the cell numbers in five different fields to obtain an average value as an indicator of cell invasive capacity.

### Statistical analysis

Data pre-processing and analysis were carried out in the R and Perl language environment. Statistical differences between the two groups were tested using the Wilcox test, correlations between the two groups were explored using Spearman’s rank correlation analysis, and ROC curves were used to assess the AUC of the different indicators. All cell experiments were performed in triplicate, and data are presented as mean ± standard deviation (SD). Cell statistical analysis was conducted using GraphPad Prism 8 software, with t-tests employed to compare differences between two groups. *P.adjust* < 0.05 was defined as statistically different.

## Results

### Generation of HCC-associated PRLs via WGCNA

**Fig. 1:**
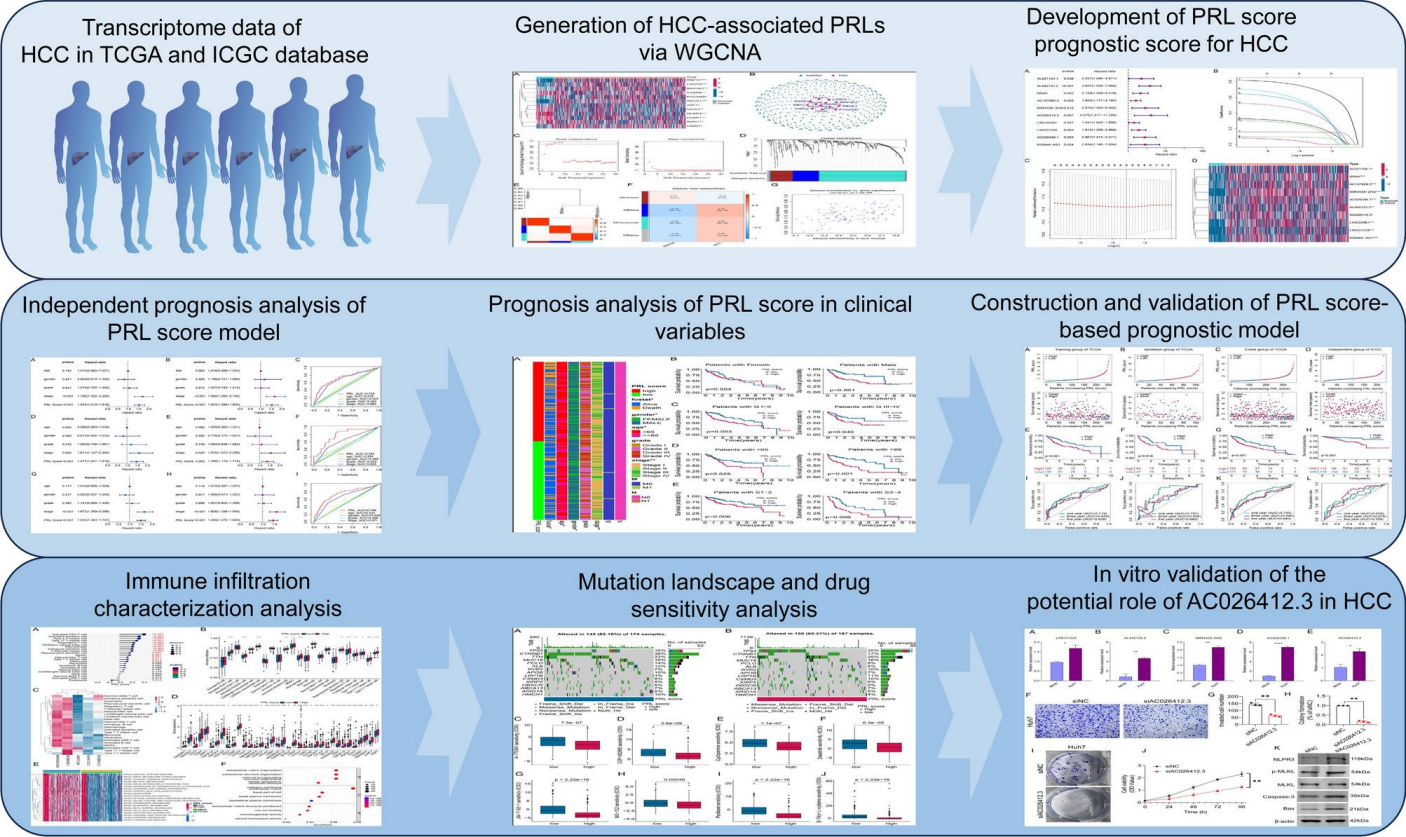
The workflow framework of this study.

**Fig. 2:**
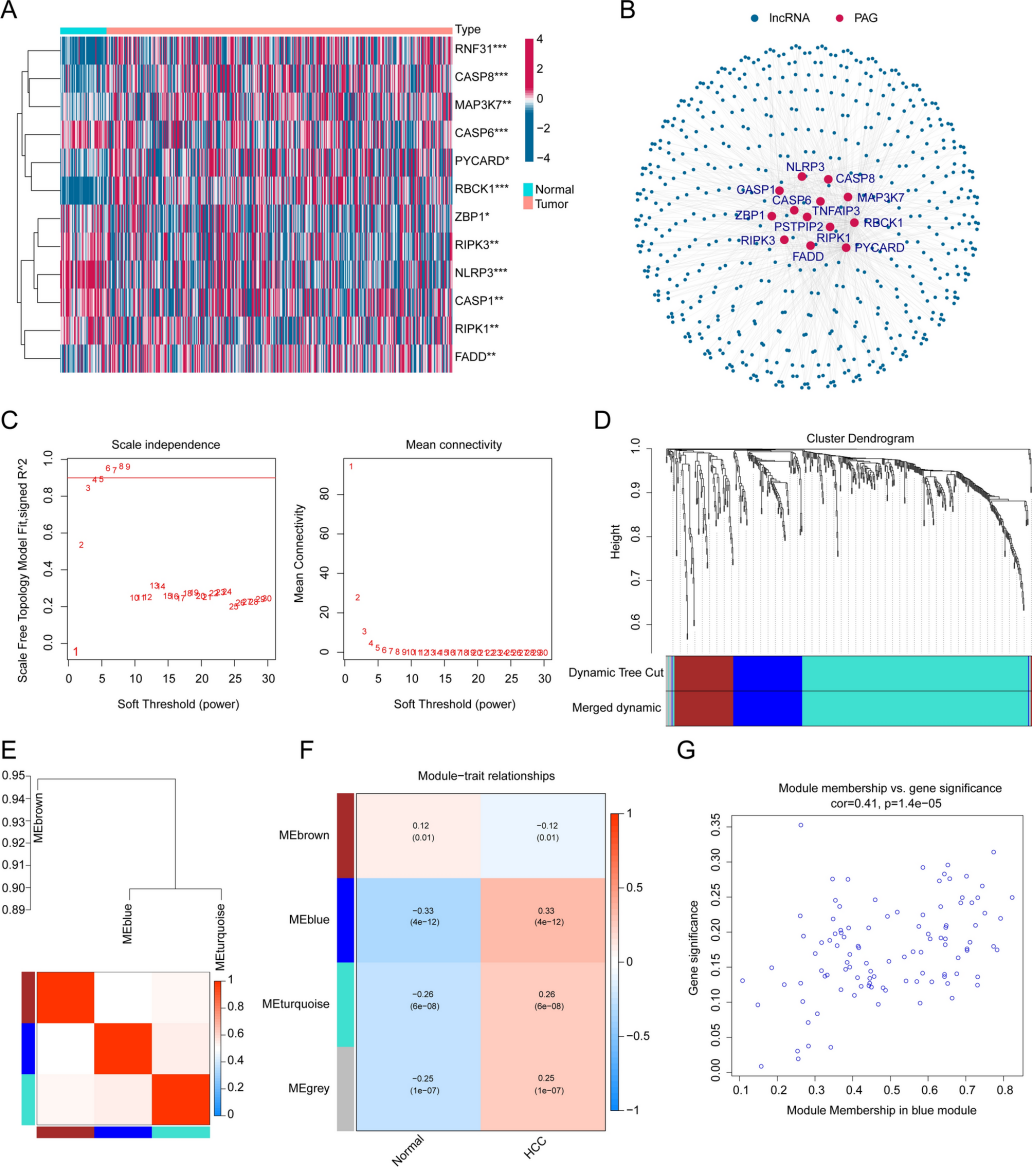
Exploration of characteristic HCC-related PRLs for HCC. (**A**) Expression profile of PAGs in normal and HCC samples. (**B**) Identification of PRLs for HCC. (**C**) The selection of soft threshold for WGCNA. (**D**) Generation of gene module by dynamic tree cut. (**E**) Relationship of gene module. (**F**) Analysis of gene modules and clinical features. (**G**) Scatter plot of module membership versus gene significance in blue module.

In this study, the analytical workflow is illustrated in Fig. 1. To determine the feature of PRLs in the progression of HCC, difference analysis was carried out the explore the expression profile of 14 PRGs in normal and HCC tissues. As displayed in Fig. 2A, we observed that the expression profile of 12 PRGs was conspicuously different in normal and HCC tissues. Under the cutoff of coefficient > 0.3, 547 lncRNAs which greatly associate with PRGs were screened as PRLs (*p* < 0.001, Fig. 2B). In order to select the HCC associated PRLs, we development a WGCNA to identify the pivotal PRLs module. In first, after the data cleansing, the samples were clustered and the outlier samples were deleted. The soft threshold (power = 4) was chosen to establish the scale-free network (R^2^ > 0.85, Fig. 2C). The cluster dendrogram displayed the height of gene module, and the cut by dynamic tree to merge the module (Fig. 2D). In each gene module, the correlation analysis showed a weak association, indicating a clear independence of gene modules (Fig. 2E). After the exploration of gene modules and clinical variables, the bule module was evaluated as the most feature module and was selected for the further analysis (Fig. 2F). The scatter plot revealed a highly association of gene significance and module membership and 105 feature HCC-associated PRLs were obtained (Fig. 2G).

### Development of PRL score for HCC-associated PRLs

**Fig. 3:**
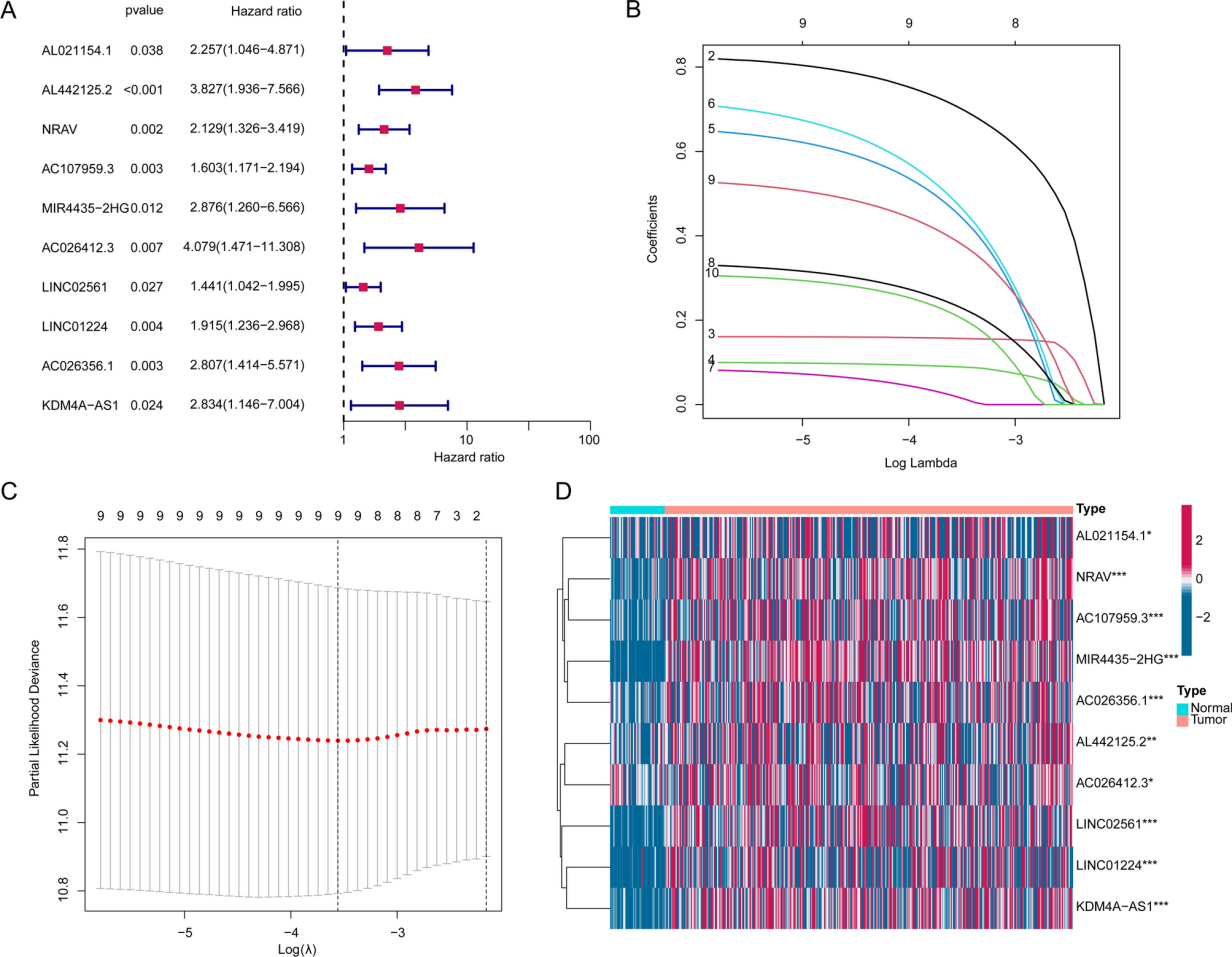
Prognostic PRLs selection for HCC. (**A**) Identification of prognostic PRLs. (**B**, **C**) Exploration of feature variables from the prognostic PRLs for HCC. (**D**) Difference analysis of prognostic PRLs in normal and HCC samples.

We further explored the prognostic value of 105 feature PRLs for HCC. On the basis of univariate Cox estimation, we acquired 10 prognostic PRLs which correlated with poor clinical prognosis (HR > 1, Fig. 3A). The LASSO analysis was carried out to select the characteristic variables for the 10 prognostic PRLs and 9 pivotal variables were collected for the further analysis (Fig. 3B, C). The heatmap plot exhibited the expression profile of 10 PRLs in normal and HCC tissues and the result illustrated that the HCC tissues had higher level of 10 prognostic PRLs (Fig. 3D). Thereafter, on the basis of multivariate Cox assessment, the PRL score of each HCC samples were estimated according to the 5 independent prognostic variables, the PRL score = 0.903 × AL442125.2 + 0.872 × MIR4435 − 2HG + 1.043 × AC026412.3 + 0.389 × LINC01224 + 0.691 × AC026356.1.

### Analysis of independent prognosis ability of PRL score

**Fig. 4:**
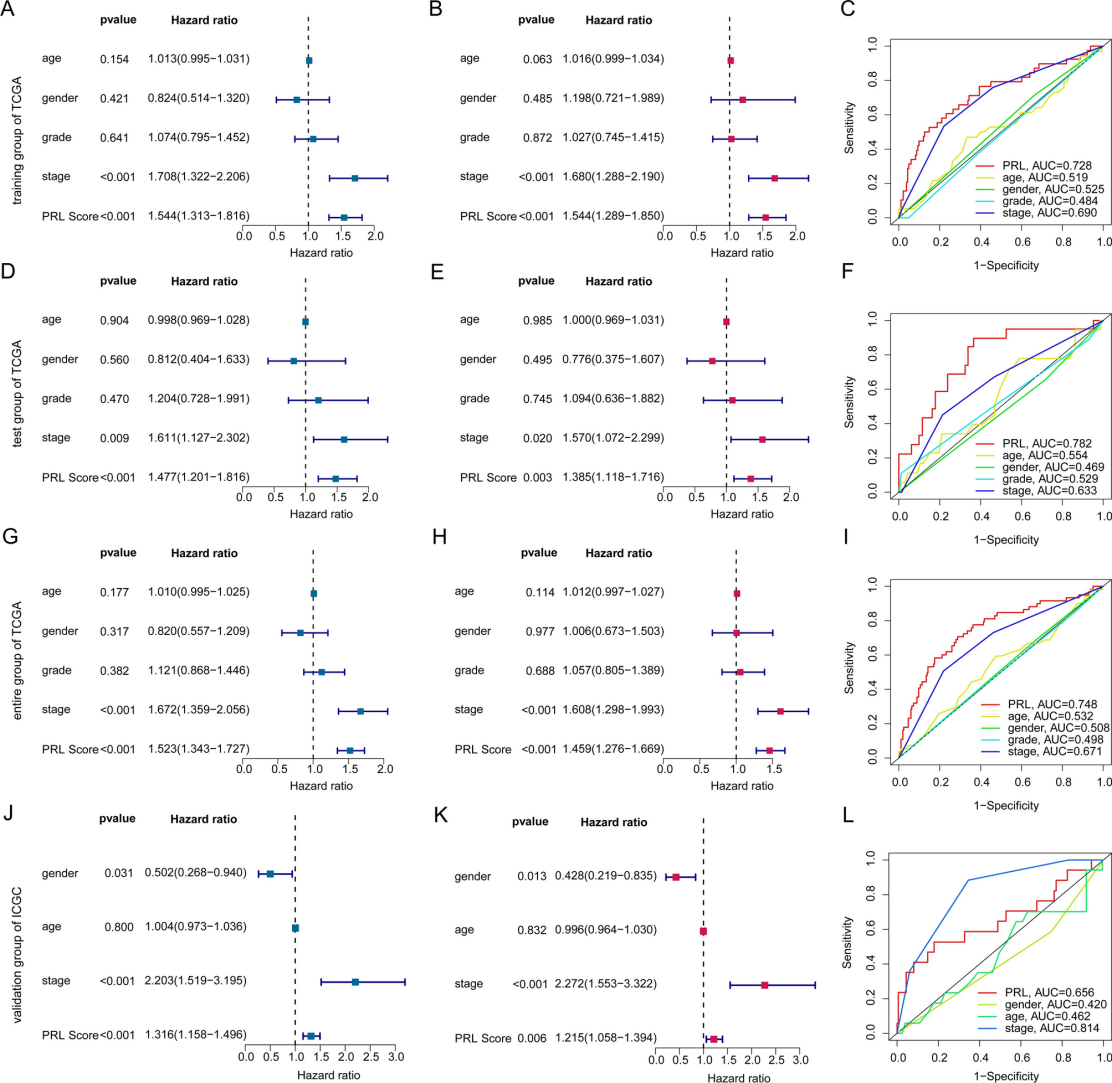
The independent prognosis evaluation of PRL score and clinical variables in TCGA and ICGC cohorts. (**A**, **B**) Univariate/multivariate Cox analysis of PRL score and clinical variables in TCGA training group. (**C**) ROC analysis of PRL score in TCGA training group. (**D**, **E**) Univariate/multivariate Cox analysis of PRL score and clinical variables in TCGA test group. (**F**) ROC analysis of PRL score in TCGA test group. (**G**, **H**) Univariate/multivariate Cox analysis of PRL score and clinical variables in TCGA entire group. (**I**) ROC analysis of PRL score in TCGA entire group. (**J**–**L**) Univariate/multivariate Cox analysis and of ROC analysis of PRL score and clinical variables in ICGC group.

According to the 5 independent prognostic variables, the HCC samples were clustered into training, test and entire groups to verify the independent prognostic value of PRL score for HCC. In addition, we utilized the ICGC dataset as an independent external validation to further elucidate the independent prognostic value of the PRL scoring system in predicting clinical survival outcomes of HCC. In the training group, the results of univariate and multivariate Cox analysis displayed that the HCC-based stage and PRL score was strongly linked with poor prognosis for HCC (Fig. 4A, B). The ROC result showed that the AUC of PRL score was 0.728, indicating a higher diagnostic power for HCC compared to other clinical features (Fig. 4C). In test and entire groups, we observed that the PRL score was clearly associated with worse clinical outcome of HCC and the AUC of PRL score in test group and entire group was 0.728 and 0.748, respectively (Fig. 4D–I). In the independent ICGC dataset, the analysis of independent prognostic value demonstrated that gender, stage, and PRL score were all associated with the clinical prognosis of HCC. Moreover, the ROC curve analysis indicated that the AUC of the PRL scoring system was 0.656 (Fig. 4J–L). Overall, these results demonstrate that the PRL score established by 5 independent prognostic variables could reflect the clinical outcome as an independent factor, implying a dependable diagnostic ability for HCC.

### Association of PRL score and clinical characteristic of HCC

**Fig. 5:**
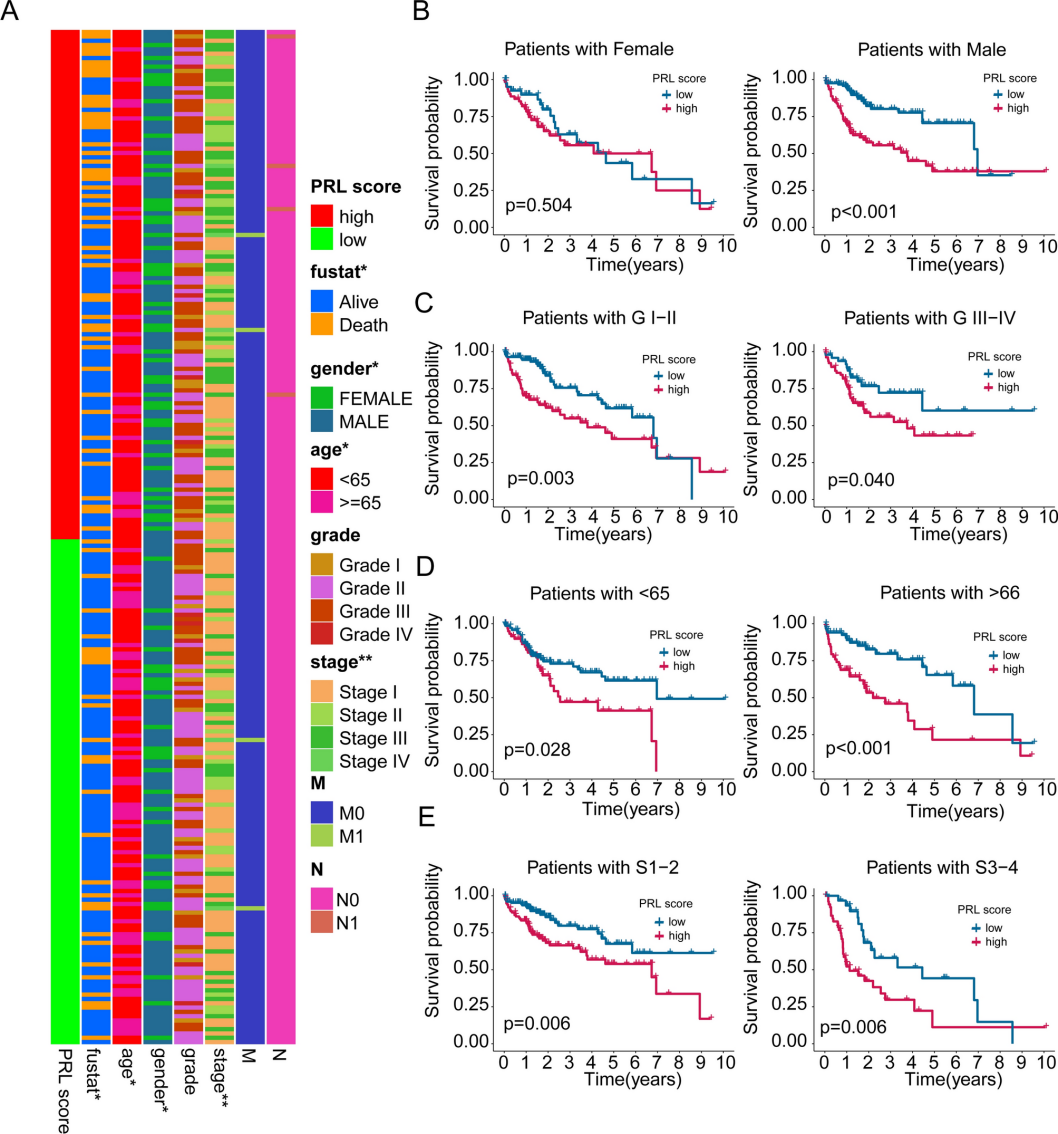
Analysis of prognostic value of PRL score in different clinical variables. (**A**) Heatmap illustrates the distribution of clinical variables in PRL score subgroups for HCC. Clinical prognosis analysis of HCC samples in the low- and high-PRL score among (**B**) gender, (**C**) grade, (**D**) age, (**E**) stage.

The relationship of PRL score and different clinical features was further explored. The heatmap diagram implied the distribution of PRL score in the different clinical features, and a noteworthy difference was observed of PRL score in clinical status, gender, age and stage (Fig. 5A). Additionally, the clinical prognosis of HCC samples with low- and high PRL score in different clinical features was further clarified. As displayed in Fig. 5B–E, the clinical outcome of HCC samples with low PRL score was better than those with high PRL score among male, grade, age, and stage, whereas the prognosis of female HCC samples with low- and high PRL score showed no difference. We further evaluated the prognostic value of the PRL scoring system across different clinicopathological characteristics in the independent external validation ICGC dataset. Differential analysis of various clinicopathological features indicated that the distribution of PRL scores differed significantly in clinical survival outcomes and stages within the ICGC dataset (Supplementary Fig. 1A). Clinical prognostic curve analysis revealed that in the clinicopathological subgroups of age ≥ 65, male, female, and stage I-II, HCC samples with low PRL scores demonstrated better survival benefits (Supplementary Figs. 1B-1G). These results illustrate that the PRL score could better interpret the clinical prognosis of HCC samples in different clinical characteristics.

### Development of PRL score-based risk model to evaluate the prognosis of HCC

**Fig. 6:**
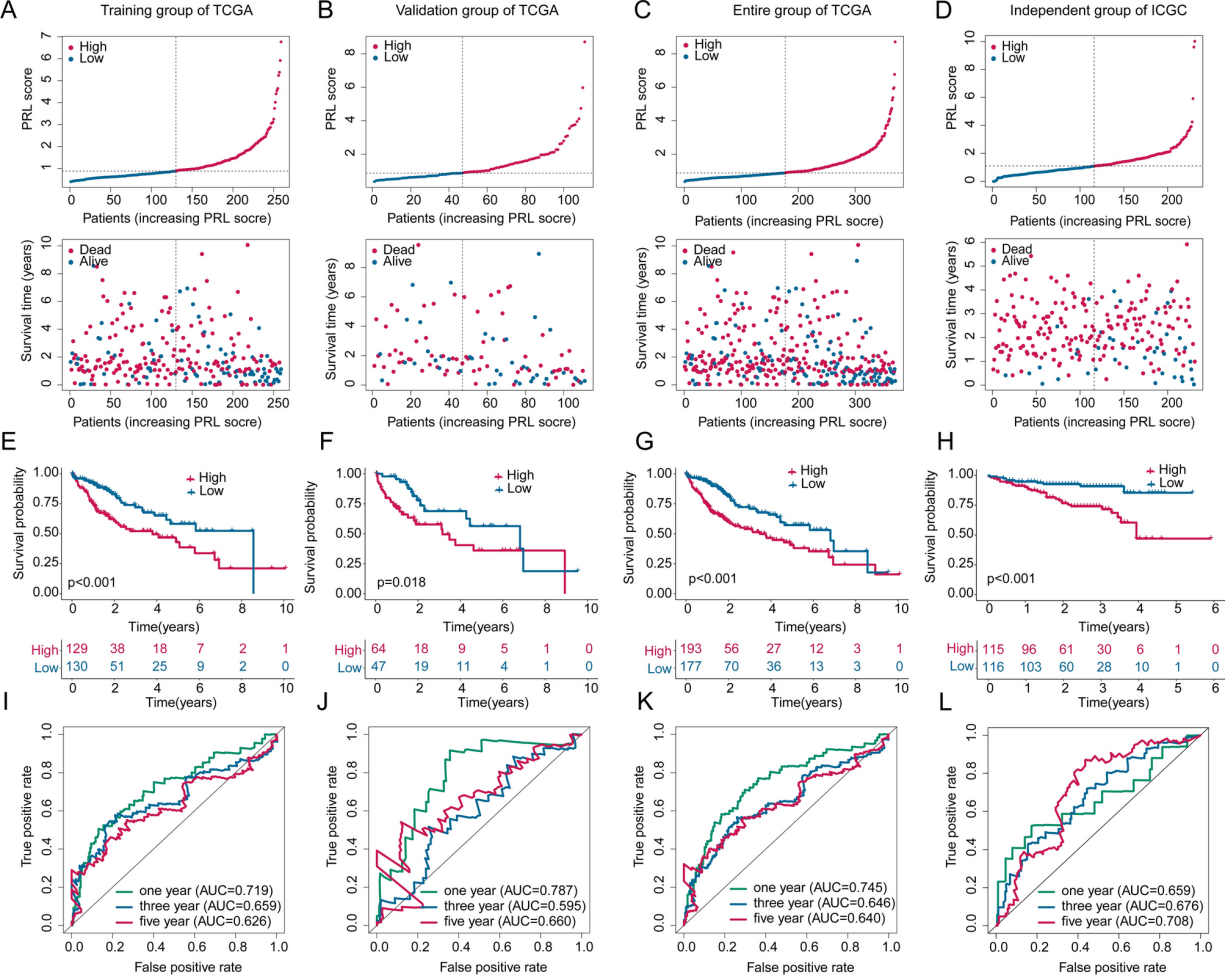
Risk model establishment of PRL score for HCC in TCGA and ICGC database. (**A**–**C**) The classification of PRL score subgroups in TCGA database. (**D**) PRL score subgroups classification of HCC in ICGC database. (**E**–**H**) Clinical prognosis estimation of HCC samples with low- and high PRL score in TCGA (training, test, entire groups) and ICGC database. (**I**–**L**) Time-dependent ROC analysis in TCGA (training, test, entire groups) and ICGC database.

To better understand the PRL score in predicting clinical outcome for HCC, we developed a novel risk model of HCC samples in TCGA database (training, test, entire groups) and ICGC database. In the 3 independent groups of TCGA database and ICGC database, the HCC samples were clustered into low- and high PRL score subgroups, and the HCC samples with high PRL score were related with worse survival time (Fig. 6A–D). The analysis of prognosis in the TCGA database (training, test, entire groups) and ICGC database implied that the clinical outcome of HCC samples with low PRL score was remarkably better compared to those high PRL score samples (Fig. 6E–H). The 1-, 3-, and 5-year AUC of time-dependent ROC curve was 0.719, 0.659, 0.626 in training group; 0.787, 0.595, 0.660 in test group; 0.745, 0.646, 0.640 in entire group, and 0.659, 0.676, 0.708 in ICGC database, respectively (Fig. 6I–L).

### Development of a nomogram diagnostic model based on the PRL scoring system and clinicopathological features in independent cohorts

**Fig. 7:**
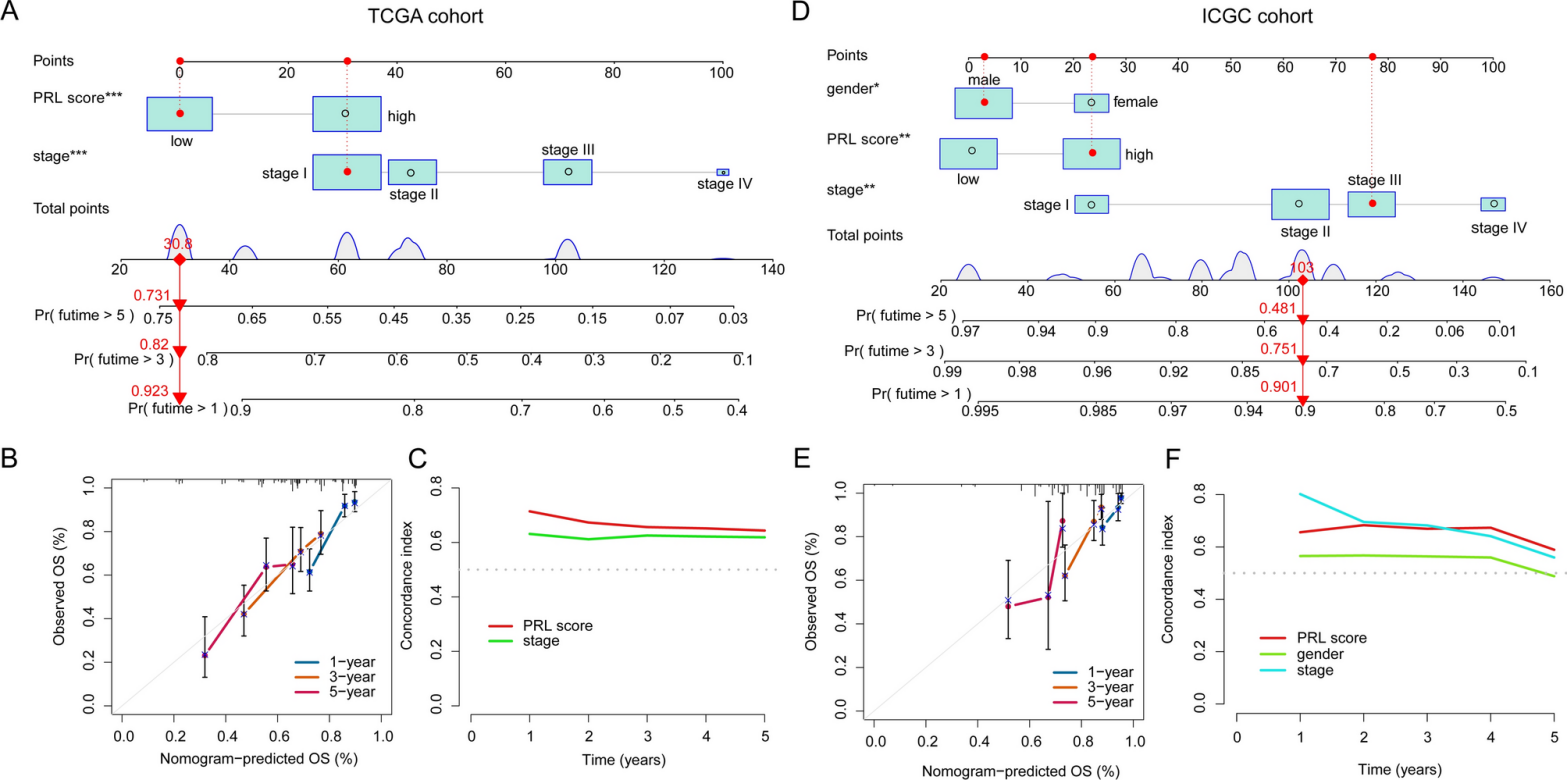
Development of a nomogram diagnostic model based on the PRL scoring system and clinicopathological variables in the TCGA and ICGC cohorts. (**A**) Construction of the nomogram diagnostic model in the TCGA cohort. (**B**, **C**) Calibration curves and concordance index analysis for different variables. (**D**) Construction of the nomogram diagnostic model in the ICGC cohort. (**E**, **F**) Calibration curves and concordance index analysis for different variables.

By integrating clinicopathological variables with independent prognostic significance and the PRL scoring model, we developed a nomogram to predict the 1-year, 3-year, and 5-year survival probabilities of HCC patients in two independent cohorts. In the TCGA cohort, the nomogram’s predicted survival probabilities aligned closely with the observed outcomes (Fig. 7A, B). Moreover, the C-index analysis confirmed that the PRL scoring model significantly surpassed the staging system in prognostic accuracy for HCC (Fig. 7C). Similarly, in the independent ICGC validation cohort, the nomogram demonstrated a high degree of concordance between predicted and actual survival probabilities. Importantly, the PRL scoring model exhibited markedly better prognostic performance for HCC compared to both gender and stage (Fig. 7D–F).

### The relationship of PRL score and immune infiltration

**Fig. 8:**
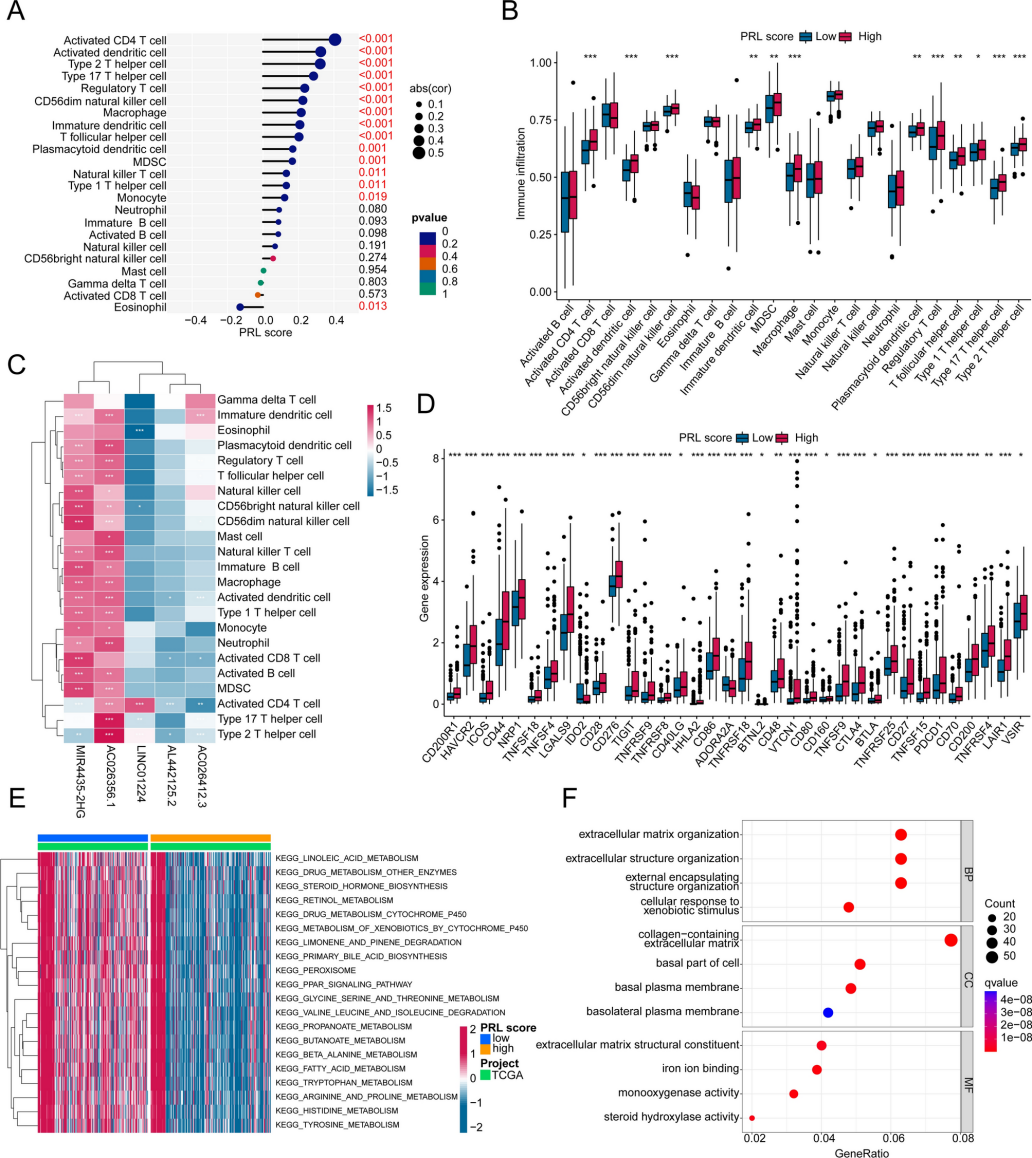
The estimation of immune infiltration and molecular biological of HCC samples in the PRL score subgroups. (**A**) Exploration of PRL score and immune infiltration. (**B**) Proportion of immune cells in PRL score subgroups. (**C**) Correlation analysis of prognostic factors and immune cell. (**D**) Expression profile of ICP in PRL score subgroups. (**E**) GSVA evaluation of KEGG terms. (**F**) GO enrichment assessment.

We further clarified the potential relationship of PRL score and immune infiltration for HCC. On the basis of correlation analysis, a remarkable association was discovered between PRL score and immune infiltration (Fig. 8A). The PRL score was negatively related to eosinophil, but positively related to CD4^+^ T cell, activated dendritic cell, type 2 T helper cell and type 17 T helper cell. As estimated by ssGSEA algorithm, a noteworthy gap was observed in immune infiltration of HCC samples in low- and high PRL score subgroups, such as CD4^+^ T cell, activated dendritic cell, type 2 T helper cell and type 17 T helper cell (Fig. 8B). Moreover, the correlation analysis of 5 prognostic PRLs and immune infiltration also displayed a clear association (Fig. 8C). The difference analysis exhibited a significant distinction of immune checkpoint proteins (ICPs) in PRL score subgroups, such as CD44, CD276, HHLA2, BTLA and CTLA4 (Fig. 8D). Gene set variation analysis (GSVA) assessment result suggested that a series of metabolism related pathways were greatly downregulated of HCC samples with high PRL score, involving in alanine metabolism, fatty acid metabolism, tryptophan metabolism and histidine metabolism (Fig. 8E). The GO enrichment analysis revealed that extracellular matrix organization, extracellular structure organization and collagen-containing extracellular matrix may act pivotal function in the tumorigenesis of HCC (Fig. 8F). Collectively, our results demonstrate that the prognosis model of PRL score is associated with immune status and could evaluate the immune infiltration of HCC samples in PRL score subgroups.

### Evaluation of mutation landscape and chemotherapy drug prediction

**Fig. 9:**
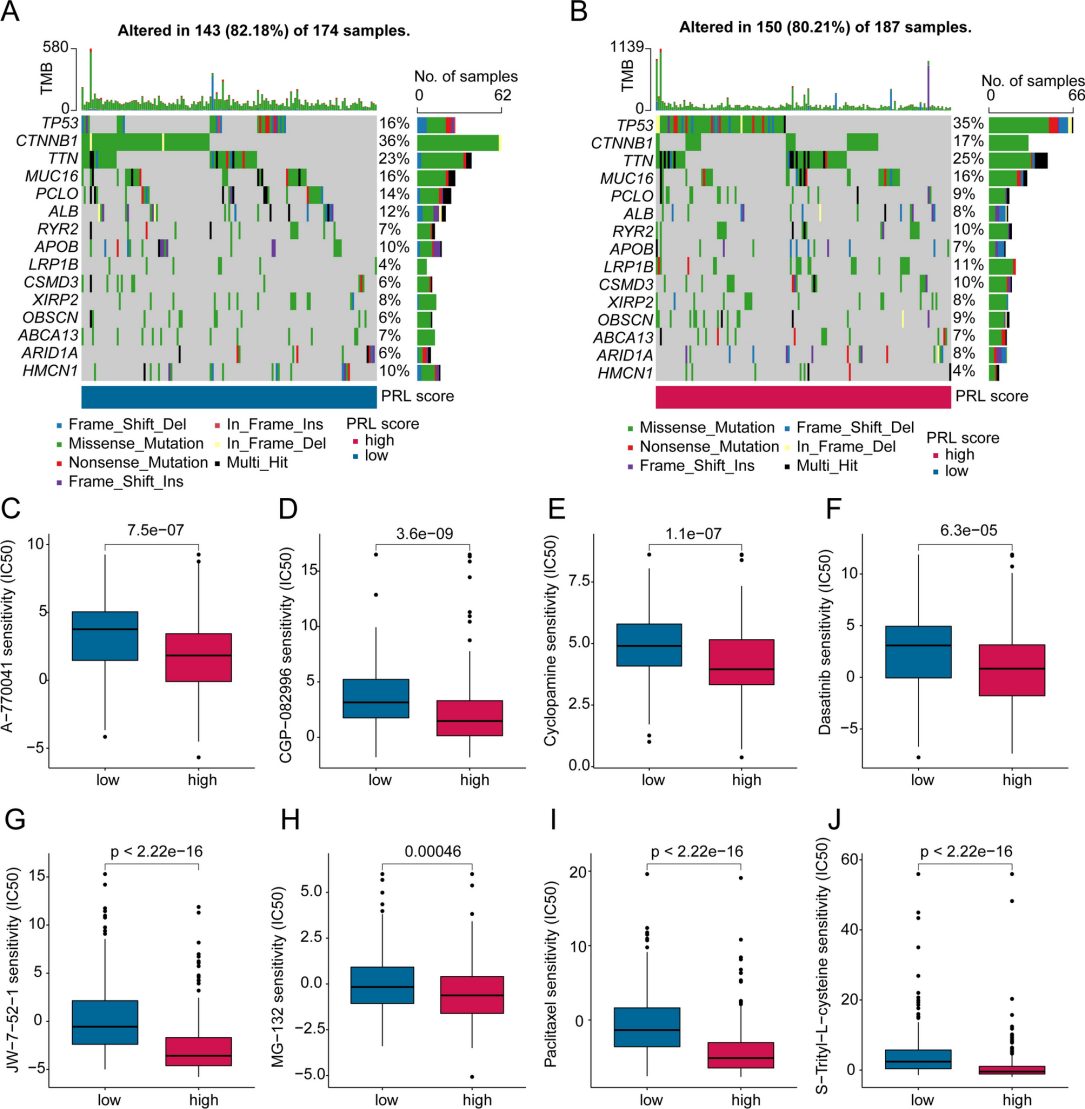
Somatic mutation landscape and chemotherapy drug prediction. (**A**, **B**) Somatic mutation frequency in PRL score subgroups. (**C**–**J**) IC50 distribution of chemotherapy drug in PRL score subgroups.

The landscape of somatic mutation of HCC samples in PRL score subgroups was further explored. The TMB results indicated 143 (82.18%) of 174 HCC samples was altered in the low PRL score group and 150 (80.21%) of 187 HCC samples was altered in high PRL score group (Fig. 9A, B). Compared to high PRL score group, the mutation frequency of TP53, TTN, RYR2 were lower in the low PRL score group, whereas the mutation frequency of CTNNB1, PCLO and ALB were higher in the low PRL score group. Additionally, we further explored some chemotherapy drugs which may benefit for the treatment of HCC based on GDSC database. As shown in Fig. 9C–J, we observed that the IC50 of A-770041, CGP-082996, cyclopamine, dasatinib, JW-7-52-1, MG-132, paclitaxel and S-Trityl-L-cysteine were higher in low PRL score group. In summary, these results illustrate that the PRL score could reflect the mutation landscape and potential chemotherapy drug response of HCC samples, providing a newly perspective for the individualized treatment of HCC.

### Knockdown of AC026412.3 inhibits HCC progression and invasion

**Fig. 10:**
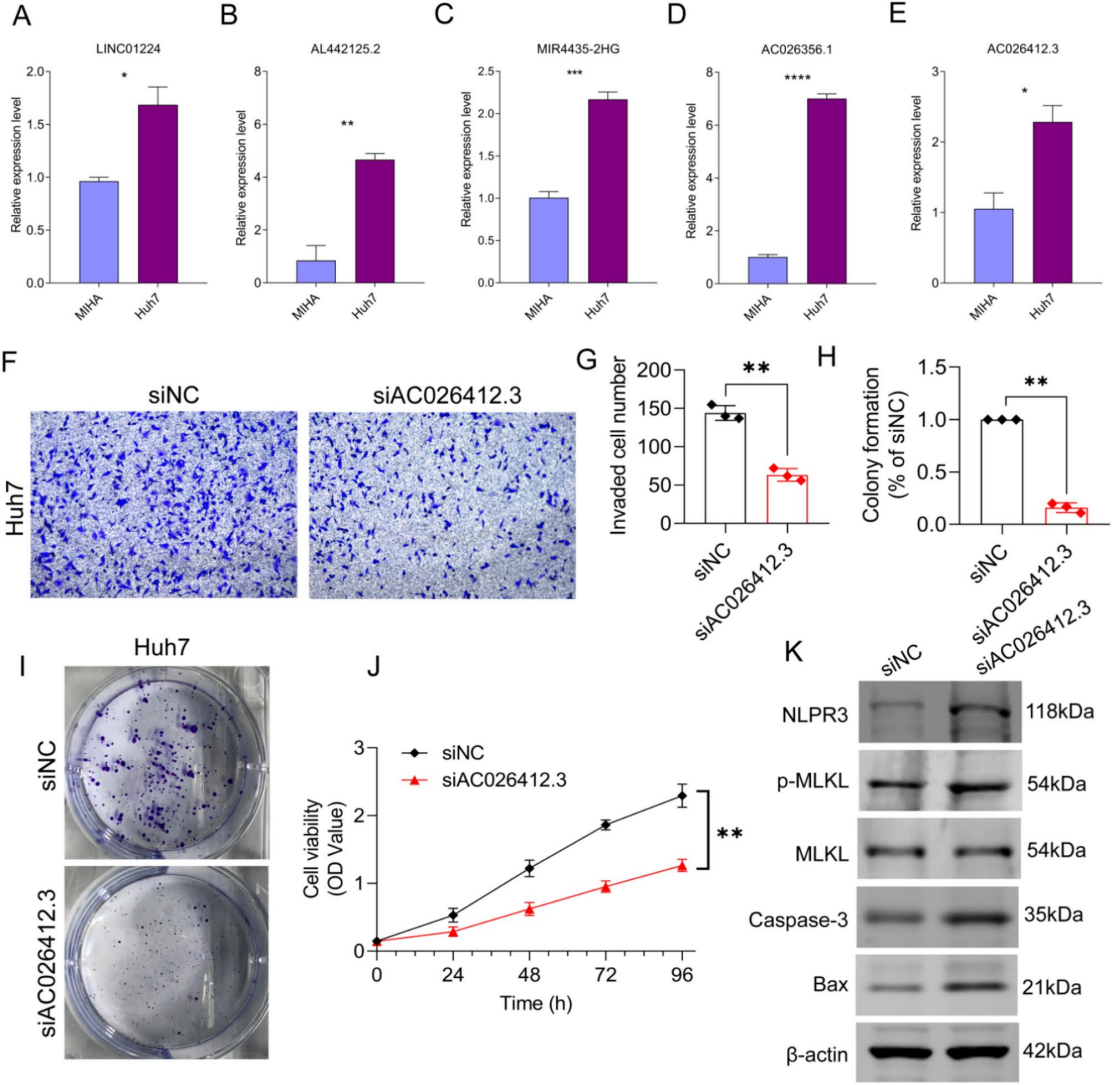
Knockdown of AC026412.3 inhibits HCC progression and invasion. (**A**–**E**) The mRNA expression levels of 5 prognostic related PRL in MIHA and Huh7 cell lines. (**F**, **G**) Transwell analysis to assess the effects of AC026412.3 interference on cell invasion abilities (n = 3). (**H**, **I**) Cell colony formation assay (n = 3). (**J**) Cell viability assay of Huh7 cell lines treated with the control vector (siNC) and siAC026412.3 (n = 3). (**K**) Protein expressions of Caspase-3, Bax, NLRP3, MLKL and p-MLKL after interference with AC026412.3. Data are presented as mean ± SD. Statistical significance: *p < 0.05; **p < 0.01; ***p < 0.001.

In our in vitro cell experiments, we validated the consistency of prognosis-related PRLs with data from the TCGA database. As shown in Fig. 10A–E, the expression levels of AC026356.1, AC026412.3, AL442125.2, LINC01224, and MIR4435-2HG were significantly higher in the Huh7 cell line compared to the MIHA cell line. Based on the risk coefficients for these five PRLs in the clinical prognosis of HCC, we found that AC026412.3 had the highest risk coefficient, suggesting it may be a key factor influencing HCC prognosis. To investigate whether AC026412.3 affects HCC progression, we transfected the Huh7 HCC cell line with AC026412.3 siRNA. Transwell assay results showed that knockdown of AC026412.3 significantly reduced the number of Huh7 cells migrating through the lower chamber (Fig. 10F, G). Additionally, we observed that knockdown of AC026412.3 markedly suppressed the colony-forming ability of Huh7 cells (Fig. 10H, I). Lastly, using an MTT assay to assess cell viability at 24, 48, 72, and 96 h, we found that interference with AC026412.3 significantly reduced the proliferative capacity of Huh7 cells (Fig. 10J). In order to further verify the effect of interference with AC026412.3 on PANoptosis, we detected the expression of a variety of cell death related proteins by western blot. The results showed that the expressions of Caspase-3, Bax, NLRP3 and p-MLKL were significantly increased after interference with AC026412.3 (Fig. 10K). These results suggest that interfering with AC026412.3 can play a killing role in tumor by promoting cell PANoptosis.

## Discussion

In this study, we explored the relationship between PRLs and HCC and established a prognostic model. After the reliability of the prognostic model was verified, immunoinfiltration and drug sensitivity analysis provided the basis for clinical personalized risk stratification of HCC. PCR experiment further verified the reliability of bioinformatics results. The impact of one of the screened PRLs, AC026412.3, on HCC progression and invasion was further confirmed, which gave evidence of the reliability of the bioinformatic research.

There have been several reports on PANoptosis-related lncRNAs as prognostic markers in cancers, including pancreatic cancer and lung cancer^21,22^ . The specific mechanisms may be related to the differences in the immune microenvironment and the response to anticancer drugs. However, the selected lncRNAs are not consistent across different types of tumors, suggesting the complexity of the role of PANoptosis in various cancer types^21,22^. Among the selected PRLs, AC026412.3, AC026356.1 and LINC01224 were only reported to have prognostic correlation in HCC, and the mechanism involved in tumorigenesis was not discussed in depth^23–25^. MIR4435-2HG is a key molecule in the progression of multiple tumors^26^. MIR4435-2HG can inhibit apoptosis, increase cell proliferation, invasion and migration, and enhance epithelial-to-mesenchymal transition (EMT). In addition, MIR4435-2HG can regulate multiple signaling pathways including Wnt, PI3K/AKT, and MAPK/ERK, thereby regulating the biological activity of a variety of tumors, including HCC^26^. Hepatic stellate cell (HSC) activation is a key event in the development of hepatic fibrosis and HCC. MIR4435-2HG is involved in the promotion of malignant behavior of HCC by HSC and may be a potential therapeutic target^27^. Although we found AC026412.3 had the highest risk coefficient among all the PRLs, we were not able to give a possible explanation for this phenomenon, as no detailed impact of AC026412.3 on carcinogenesis progress. Since we have made preliminary in vitro evidence, further molecular mechanism research would be interest.

One interesting observation we made in this study was that PRL scores were associated with prognosis in male with HCC but not in female with HCC. There are gender differences in the occurrence and development of HCC, and the incidence of HCC in men is four times that in women^28^. Although the specific mechanism is not clearly understood, there are some possible explanations. There is evidence that estrogen receptors inhibit NF-κB binding activity, thereby inhibiting the tumor-forming process^29^. Estrogen therapy reduced the incidence and metastasis of HCC, and increased susceptibility to HCC after oophorectomy in female mice^30^. In addition, estrogen may play an inhibitory role in sex differences in hepatocellular carcinoma through microRNAs, DNA repair, and obesity-related pathways^31–33^. In contrast, overexpression of androgens enhances the growth and invasion of HCC cells, as well as the initiation of HCC in vivo^34^. During hormone regulation, several hormone related targets such as 17-β-estradiol, estrogen receptor and its associated PARP1 have been reported to be associated with programmed cell death^33,35,36^ In addition, other HCC targets have been reported to be sex-dependent and sensitive to specific risk factors^37^. Our evidence suggests a potential relationship between the prognostic value of PRLs and the gender of HCC patients, and the specific mechanisms need to be further explored.

Our results show that high PRL scores are associated with higher Tumor Protein p53 (TP53) and lower Catenin Beta 1 (CTNNB1) mutation rates. The combination of TP53 and CTNNB1 gene mutations facilitates early detection and recognition of HCC^38^. TP53, as a widely recognized poor prognostic factor, was positively associated with PRL score and poor prognosis. Chronic infection with hepatitis virus and exposure to dietary aflatoxins, two major triggers of HCC, are both associated with TP53 mutations in HCC molecular pathogenesis^39^. CTNNB1 plays a key role in hepatocyte adhesion and Wnt signaling pathways^40^. CTNNB1 mutation has been reported as an indicator of good prognosis of liver cancer and a new target for treatment^41^. The mechanism of CTNNB1 mutation in HCC is complex, and it has both antitumor and metastasis promoting effects^42^. At the same time, T-box transcription factor 3 (TBX3) induced by CTNNB1 mutant can inhibit the growth of HCC and inhibit the YAP/TAZ oncogene^43^. In addition, there is evidence that CTNNB1 mutations are associated with a longer recurrence interval and a higher density of CD8^+^ T lymphocytes in the tumor center^44^. However, evidence to the contrary of these results includes: HCC patients with CTNNB1 mutations benefit less from ICI^45,46^; The activation of CTNNB1 expression can increase the stemness of HCC through lncRNA DANCR^47^. Our results show that CTNNB1 mutations appear to be associated with a better prognosis for HCC. Limited to the number of specimens, further multicenter studies and in-depth mechanism studies are helpful to specifically analyze the different effects of CTNNB1 on HCC patients.

The nomogram is an easy-to-use prognosis model for cancer patients that can help physicians make more accurate clinical decisions^48,49^. In addition to AFP, tumor stage, vascular infiltration, age, and other common prognostic risk factors, increasing number of genetic signatures have been added to build the nomogram model for HCC^50^. Our results showed that the PRL scoring model we developed had higher prognostic efficacy in comparison to neoplasm staging. There is no consensus on which prognostic risk stratification system is most effective for HCC prognosis, as different risk stratification methods have different efficacies in different HCC subgroups^51,52^. Therefore, the PRL scoring model still need to be subjected to more precise clinical analysis in the clinic to identify the most beneficial patient groups.

Due to the limitation of key clinical or molecular information in public databases, we were unable to extract studies on the mechanism of PANoptosis process in HCC. In addition, public databases are unavoidably biased by region and ethnicity. Future multi-center, more in-depth studies will help to further reveal the important value of PANoptosis in HCC. As a bioinformatics research-based study, the results presented in this paper are mostly correlation analysis rather than causation analysis. It is of potential clinical value to further explore the role of the selected PRL in HCC through in vitro experiments and explain whether there is a causal relationship with the occurrence and development of HCC.

## Supplementary Information

Supplementary Information 1.

Supplementary Information 2.

Supplementary Information 3.

Supplementary Information 4.

Supplementary Information 5.

Supplementary Information 6.

Supplementary Information 7.

## Data Availability

The datasets used during the current study are available from the corresponding author upon reasonable request.

## Abbreviations

AFP: Alpha-fetoprotein
ATCC: American type culture collection
AUC: Area under curve
CASP8: Caspase-8
C-index: Concordance index
DMEM: Dulbecco’s modified eagle medium
DMSO: Dimethyl sulfoxide
EMT: Epithelial-to-mesenchymal transition
FBS: Fetal bovine serum
FPKM: Fragments per Kilobase million
GAPDH: Glyceraldehyde 3-phosphate dehydrogenase
GDSC: Genomics of drug sensitivity in cancer
GO: Gene ontology
GSVA: Gene set variation analysis
HCC: Hepatocellular carcinoma
IC50: Half maximal inhibitory concentration
ICGC: International cancer genome consortium
ICP: Immune checkpoint
ICI: Immune checkpoint inhibitors
KEGG: Kyoto encyclopedia of genes and genomes
LASSO: Least absolute shrinkage and selection operator
lncRNA: Long non-coding RNA
MAF: Mutation annotation format
MTT: 3-(4,5-Dimethylthiazol-2-yl)-2,5-Diphenyltetrazolium Bromide
NLRP3: NOD-, LRR- and pyrin domain-containing protein 3
PANoptosome: Protein complex involved in PANoptosis
PBS: Phosphate-buffered saline
PRG: PANoptosis-related gene
PRLs: PANoptosis-related lncRNAs
RCD: Regulated cell death
RIP1: Receptor-interacting serine/threonine-protein kinase 1
ROC: Receiver operating characteristic
RT-qPCR: Real-time quantitative PCR
siRNA: Small Interfering RNA
ssGSEA: Single sample gene set enrichment analysis
TCGA: The cancer genome atlas
TKI: Tyrosine kinase inhibitors
TMB: Tumor mutation burden
WGCNA: Weighted gene co-expression network analysis
ZBP1: Z-DNA binding protein 1
