# Supplementary figures and images for: Identification and validation of PANoptosis-related LncRNAs prognosis system in hepatocellular carcinoma
Source: Sci Rep. 2025 Feb 19;15:6030. doi: 10.1038/s41598-025-90498-y (PMC11840146; doi:10.1038/s41598-025-90498-y)

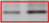

BAX

Supplement: Supplementary file 1 — Supplementary Information 1. [file 41598_2025_90498_MOESM1_ESM.pdf]

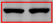

beta-actin

Supplement: Supplementary file 2 — Supplementary Information 2. [file 41598_2025_90498_MOESM2_ESM.pdf]

Caspase-3

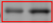

Supplement: Supplementary file 3 — Supplementary Information 3. [file 41598_2025_90498_MOESM3_ESM.pdf]

MLKL

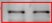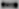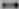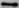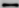

Supplement: Supplementary file 4 — Supplementary Information 4. [file 41598_2025_90498_MOESM4_ESM.pdf]

P-MLKL

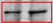

Supplement: Supplementary file 5 — Supplementary Information 5. [file 41598_2025_90498_MOESM5_ESM.pdf]

# NLRP3

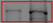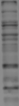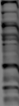

Supplement: Supplementary file 6 — Supplementary Information 6. [file 41598_2025_90498_MOESM6_ESM.pdf]
