# Supplementary material for: Identification and validation of PANoptosis-related LncRNAs prognosis system in hepatocellular carcinoma
Source: Sci Rep. 2025 Feb 19;15:6030. doi: 10.1038/s41598-025-90498-y (PMC11840146; doi:10.1038/s41598-025-90498-y)
Supplement: Supplementary file 7 — Supplementary Information 7. [file 41598_2025_90498_MOESM7_ESM.docx]

**Supplementary Figure 1. Prognostic value evaluation of the PRL scoring system across different clinicopathological characteristics in the ICGC dataset.** (A) Differential analysis of PRL scores among various clinicopathological characteristics. (B-G) Prognostic value assessment of PRL scores within different clinicopathological subgroups.


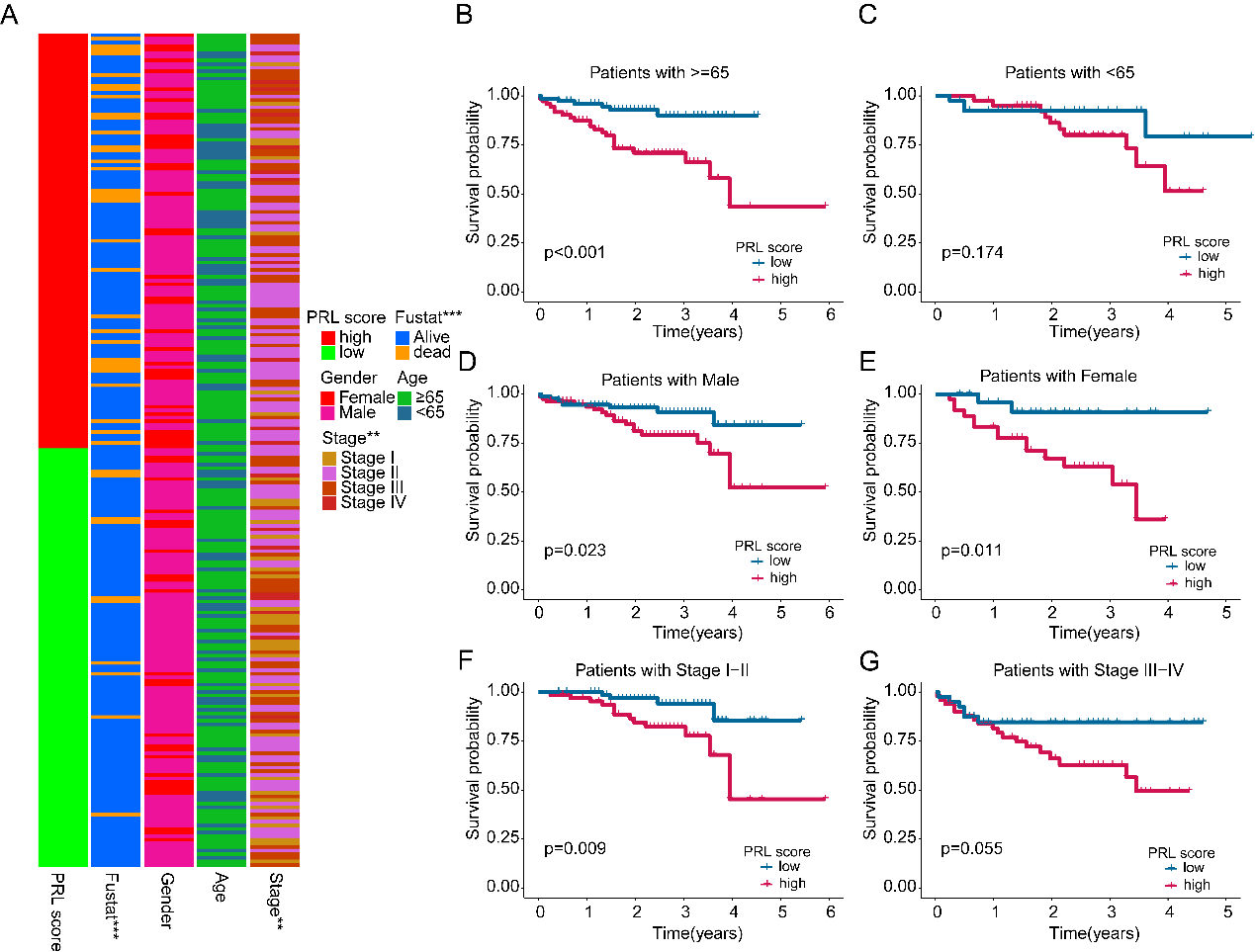


| Supplementary table 1. The gene list of PRGs. |
| --- |
| ZBP1 |
| NLRP3 |
| RIPK1 |
| RIPK3 |
| CASP1 |
| CASP6 |
| CASP8 |
| PYCARD |
| FADD |
| MAP3K7 |
| TNFAIP3 |
| RNF31 |
| RBCK1 |
| PSTPIP2 |

Supplementary table 2. Identification of PRL in HCC.

| LINC01063 |
| --- |
| MCM3AP-AS1 |
| AP000759.1 |
| TRIM52-AS1 |
| AC245060.2 |
| AC023302.1 |
| GPRC5D-AS1 |
| AL021154.1 |
| AC018755.4 |
| AC103808.3 |
| AC004596.1 |
| Z99943.1 |
| LINC02335 |
| AL117335.1 |
| TDRKH-AS1 |
| AC109460.2 |
| AC136604.2 |
| AC026333.4 |
| DHCR24-DT |
| AC138230.1 |
| LINC02313 |
| AL442125.2 |
| AC002094.4 |
| LINC01447 |
| AC074032.1 |
| AC006033.2 |
| AC005775.1 |
| NFYC-AS1 |
| LINC01004 |
| AC034213.1 |
| LINC01952 |
| AC079807.1 |
| CAMTA1-DT |
| AC004263.1 |
| AC007292.1 |
| AC110995.1 |
| AL117379.1 |
| AC124016.1 |
| AC145423.2 |
| AC116913.1 |
| LINC02139 |
| PAXIP1-AS2 |
| AC023043.4 |
| AC132872.2 |
| AC016876.3 |
| AC022146.2 |
| ITGA6-AS1 |
| AC010976.1 |
| AP003392.1 |
| AL133215.2 |
| ZFAS1 |
| AP007216.2 |
| AC131009.3 |
| DLG5-AS1 |
| AC142472.1 |
| AP002761.1 |
| MANEA-DT |
| AP001527.2 |
| AC011632.1 |
| RFX3-AS1 |
| AP001107.9 |
| AL592211.1 |
| SNHG8 |
| LINC02506 |
| AC067852.2 |
| AC016394.1 |
| AGBL5-AS1 |
| AC020661.1 |
| PSMB8-AS1 |
| AL133215.3 |
| TSPOAP1-AS1 |
| AC074212.1 |
| AL022341.1 |
| THAP9-AS1 |
| LINC01948 |
| LINC01876 |
| DANCR |
| AC027281.1 |
| AL354811.1 |
| AL137003.1 |
| AC132872.3 |
| PPP4R1-AS1 |
| AL133297.1 |
| AC092354.2 |
| AC027271.1 |
| AC091057.1 |
| AC137630.3 |
| AC090164.2 |
| SNHG25 |
| AC099791.2 |
| CPNE8-AS1 |
| AC092171.5 |
| AC004854.2 |
| AC015912.3 |
| LINC00924 |
| AC005586.1 |
| AP001062.1 |
| IDH1-AS1 |
| AL021707.1 |
| AL445228.2 |
| NRAV |
| AC008105.3 |
| AC010331.1 |
| AC009090.6 |
| AC114489.1 |
| G2E3-AS1 |
| AC087741.2 |
| AC092574.1 |
| MIR3142HG |
| AL591895.1 |
| AC002550.2 |
| PRRT3-AS1 |
| AC009275.1 |
| AC003070.1 |
| AC002116.2 |
| AC036176.1 |
| AL121832.2 |
| LINC01446 |
| AC010969.2 |
| AL353801.3 |
| HCP5 |
| AL139289.1 |
| AC025048.4 |
| AC009686.2 |
| AL355297.2 |
| ARF4-AS1 |
| LINC00539 |
| AC016575.1 |
| AC008915.2 |
| AC109460.1 |
| AC117386.2 |
| AC026369.3 |
| AC005224.2 |
| AC104667.2 |
| AC005899.7 |
| AC063948.1 |
| LINC00339 |
| LINC02195 |
| AL357054.4 |
| AC009271.1 |
| AC091153.3 |
| AC018529.1 |
| AC131097.3 |
| TMCC1-AS1 |
| LINC02416 |
| AC108134.4 |
| NCK1-DT |
| LINC00628 |
| PRKCZ-AS1 |
| AC092118.1 |
| AC010255.2 |
| AC104506.1 |
| SNHG6 |
| AC018690.1 |
| LINC00519 |
| AP003065.1 |
| AC090589.3 |
| AL035045.1 |
| AC073655.2 |
| TBC1D8-AS1 |
| LINC00648 |
| AC120053.1 |
| AC108134.3 |
| LINC01605 |
| AL355488.1 |
| AC064807.4 |
| AC034236.2 |
| AC129507.1 |
| SNHG9 |
| LINC01857 |
| AP002954.1 |
| AC009005.1 |
| AL008635.1 |
| AL157813.1 |
| AC005261.1 |
| AL096828.3 |
| EPB41L4A-DT |
| AC098484.4 |
| AC010247.1 |
| MINCR |
| HNF1A-AS1 |
| LINC02084 |
| AC009407.1 |
| MIR4458HG |
| PRKCQ-AS1 |
| AC005899.6 |
| AL355512.1 |
| AC009120.2 |
| DDX39B-AS1 |
| AP002387.1 |
| AP001505.1 |
| AC107959.3 |
| NDUFB2-AS1 |
| AL031985.3 |
| AC138207.2 |
| AL365330.1 |
| AC093673.1 |
| SLC12A9-AS1 |
| AL606468.1 |
| FBXL19-AS1 |
| AC090517.5 |
| AL732292.2 |
| AC007639.1 |
| AC120498.4 |
| AC015802.5 |
| AC107959.1 |
| HCG11 |
| AC010642.2 |
| Z98752.4 |
| UBR5-AS1 |
| AC073332.1 |
| AC072039.2 |
| AC027031.2 |
| AC026740.1 |
| CAHM |
| AL080317.2 |
| AC073352.1 |
| AC116407.1 |
| HMGN3-AS1 |
| LINC00265 |
| ZSCAN16-AS1 |
| AC106900.1 |
| AC079015.1 |
| AC034198.2 |
| AC004801.6 |
| AL158071.2 |
| AL021368.2 |
| AC002553.2 |
| PRR34-AS1 |
| SNHG11 |
| AC132807.2 |
| AC092614.1 |
| LINC01615 |
| ASH1L-AS1 |
| AC008738.2 |
| LINC02100 |
| AC011247.1 |
| CCDC18-AS1 |
| AC120349.1 |
| AC005332.3 |
| AC093249.6 |
| AC010615.2 |
| AP002360.1 |
| AC073655.1 |
| HCG15 |
| AC004000.1 |
| AL451042.2 |
| MIR4435-2HG |
| LINC01485 |
| AC008946.1 |
| MAPKAPK5-AS1 |
| AC145285.2 |
| AC116025.2 |
| AC108134.2 |
| AP001189.3 |
| AC008759.3 |
| AC245100.7 |
| AL136295.6 |
| AC005332.4 |
| LINC01194 |
| AP001469.2 |
| AC025181.2 |
| AC133552.5 |
| ZNF529-AS1 |
| AC018529.2 |
| AC027688.1 |
| AC010333.2 |
| LINC02490 |
| LINC01956 |
| AL132989.1 |
| AC083862.2 |
| AC011899.2 |
| FAM30A |
| AL157904.1 |
| AC100847.1 |
| LINC00853 |
| AL138966.2 |
| AC011294.1 |
| AP001189.1 |
| AC103706.1 |
| AC006960.3 |
| AC002401.4 |
| HAND2-AS1 |
| LINC00926 |
| AC012236.1 |
| LINC02245 |
| AC092115.3 |
| LINC02446 |
| C2-AS1 |
| AC078883.1 |
| LINC01311 |
| AC107294.2 |
| AC008610.1 |
| LENG8-AS1 |
| AC004812.2 |
| AL035661.1 |
| AC026412.3 |
| ACAP2-IT1 |
| MANCR |
| PRR7-AS1 |
| AL031673.1 |
| GMDS-DT |
| MIR600HG |
| LINC01679 |
| AC010618.3 |
| AL359878.2 |
| AC020765.2 |
| LINC01943 |
| AC027796.4 |
| AL121658.1 |
| AL354950.1 |
| AL590764.1 |
| AC099343.2 |
| AC009090.1 |
| AP000812.1 |
| LINC02561 |
| CDC37L1-DT |
| AC090912.1 |
| AL731684.1 |
| AC016405.3 |
| AC144831.1 |
| AC073487.1 |
| AC138207.5 |
| AC010186.3 |
| AC005104.1 |
| AL021707.6 |
| AC008972.1 |
| AL670729.3 |
| AL445524.1 |
| MAFG-DT |
| AC021078.1 |
| AL450326.1 |
| AC005776.2 |
| AC021074.3 |
| AC245297.3 |
| AC012462.3 |
| AC015922.2 |
| LINC01320 |
| AP000487.1 |
| AC245060.6 |
| AC008735.4 |
| MIR548XHG |
| AC087239.1 |
| AC040977.1 |
| ARRDC1-AS1 |
| RPARP-AS1 |
| AC015813.4 |
| LINC00996 |
| AC055855.1 |
| LINC00896 |
| TRAF3IP2-AS1 |
| USP30-AS1 |
| AL133467.4 |
| AL357140.2 |
| LINC01089 |
| AC015922.3 |
| ELF3-AS1 |
| JAKMIP2-AS1 |
| AL049838.1 |
| AC090912.2 |
| AC006059.1 |
| AC007773.1 |
| AC008735.2 |
| AC004585.1 |
| AC109479.1 |
| HOXB-AS1 |
| LINC01220 |
| AC008972.2 |
| AC096733.2 |
| AP004609.3 |
| AL110115.2 |
| TRAM2-AS1 |
| GAS5 |
| BX649632.1 |
| AC015813.1 |
| AC127537.1 |
| AL589765.6 |
| GSEC |
| C9orf139 |
| AC009065.8 |
| AL356481.1 |
| AC116366.1 |
| LINC01978 |
| MIAT |
| AC090559.1 |
| U91328.1 |
| AL023803.3 |
| MHENCR |
| AC005840.4 |
| WDFY3-AS2 |
| AL137009.1 |
| AC020658.5 |
| PVT1 |
| AC010618.2 |
| AP000240.1 |
| AC133528.1 |
| AL136419.1 |
| ZFHX2-AS1 |
| GNG12-AS1 |
| AC004241.3 |
| AC020915.2 |
| DNM3OS |
| AC116351.1 |
| AC017083.1 |
| AL603839.3 |
| LINC01589 |
| MNX1-AS2 |
| PXN-AS1 |
| AC132192.2 |
| STX17-AS1 |
| AL354892.3 |
| AL139407.1 |
| LINC01781 |
| AC005670.1 |
| TP53TG1 |
| AC099063.4 |
| AC004241.1 |
| AL354989.1 |
| DLEU1 |
| LINC01094 |
| LINC00839 |
| AC002091.1 |
| LIX1L-AS1 |
| TFAP2A-AS1 |
| TM4SF1-AS1 |
| AC087742.1 |
| AC004921.1 |
| AC008622.2 |
| SBF2-AS1 |
| AC002456.1 |
| LINC01355 |
| AL121894.2 |
| AL356652.1 |
| MIATNB |
| AL117332.1 |
| AL391056.1 |
| AC092757.3 |
| CH17-340M24.3 |
| AL049840.5 |
| AC244153.1 |
| PARD3-AS1 |
| AC046168.2 |
| AC023090.1 |
| LINC01224 |
| AC027097.2 |
| ENTPD3-AS1 |
| LINC01315 |
| AL592071.1 |
| AC109322.1 |
| AC099778.1 |
| AC009061.2 |
| YTHDF3-AS1 |
| AC087521.1 |
| AC138696.2 |
| LINC00641 |
| AL441992.1 |
| TEX41 |
| AC110285.5 |
| AC092171.4 |
| AL139123.1 |
| OSGEPL1-AS1 |
| MIR503HG |
| SNHG17 |
| SNHG12 |
| AL136985.2 |
| AC026356.1 |
| AC016773.1 |
| NARF-IT1 |
| AL031670.1 |
| LINC01730 |
| AC004825.2 |
| AL121899.1 |
| AL355388.1 |
| AC078846.1 |
| AL133410.1 |
| AL365361.1 |
| U47924.3 |
| FAM111A-DT |
| AC025569.1 |
| AC130324.1 |
| AL360007.1 |
| LINC02062 |
| LINC01186 |
| AC009065.4 |
| SUGT1P4-STRA6LP |
| SNHG3 |
| AC068756.1 |
| AC011445.1 |
| AC090409.1 |
| AC012360.3 |
| AC010883.1 |
| AC012640.1 |
| AL135924.2 |
| AC012676.4 |
| AL136295.2 |
| ELOVL2-AS1 |
| AC135050.3 |
| AL731533.2 |
| AL162411.1 |
| PICSAR |
| AC012676.1 |
| AC015849.3 |
| AL121772.3 |
| NPSR1-AS1 |
| AP003392.5 |
| LINC01678 |
| LINC00623 |
| AL353708.3 |
| AC116337.3 |
| AC025162.2 |
| GK-IT1 |
| LYRM4-AS1 |
| AC079174.2 |
| SLCO4A1-AS1 |
| PTOV1-AS2 |
| AC211433.2 |
| KDM4A-AS1 |
| AC003101.2 |
| LINC02259 |
| AC087501.4 |
| BACH1-IT1 |
| AF131215.6 |
| MELTF-AS1 |
| AP003419.3 |
| AL132655.2 |
| KMT2E-AS1 |
| TNFRSF10A-AS1 |
| AC022031.2 |
| AP001542.3 |
| AC011468.1 |
| AC004847.1 |
| AC127024.5 |
| TFAP2A-AS2 |
| AC026401.3 |
| AC015911.3 |
| AC002091.2 |
| U91319.1 |
| LINC01197 |
| AC004687.1 |
| AC093248.1 |
| AL442067.2 |
| VAC14-AS1 |
| AC011462.4 |
| AC080038.1 |
| CYTOR |
| AC092903.2 |
| AL035461.2 |

Supplementary table 3. Identification of 105 HCC-associated PRLs via WGCNA model.

| MCM3AP-AS1 |
| --- |
| AC245060.2 |
| AL021154.1 |
| AC103808.3 |
| AC004596.1 |
| LINC02335 |
| AC109460.2 |
| AL442125.2 |
| NFYC-AS1 |
| AC010976.1 |
| AP003392.1 |
| MANEA-DT |
| AC011632.1 |
| LINC02506 |
| THAP9-AS1 |
| LINC01948 |
| LINC01876 |
| AC132872.3 |
| AC091057.1 |
| NRAV |
| AC009090.6 |
| G2E3-AS1 |
| AC003070.1 |
| LINC01446 |
| AC109460.1 |
| LINC00339 |
| AC009271.1 |
| NCK1-DT |
| PRKCZ-AS1 |
| AC092118.1 |
| AC018690.1 |
| AP003065.1 |
| AC090589.3 |
| TBC1D8-AS1 |
| LINC00648 |
| AL355488.1 |
| AC098484.4 |
| AC009120.2 |
| AC107959.3 |
| AL365330.1 |
| FBXL19-AS1 |
| UBR5-AS1 |
| HMGN3-AS1 |
| LINC00265 |
| AL021368.2 |
| AC132807.2 |
| AC092614.1 |
| LINC02100 |
| CCDC18-AS1 |
| AC010615.2 |
| HCG15 |
| MIR4435-2HG |
| LINC01194 |
| AP001469.2 |
| LINC01956 |
| AL132989.1 |
| C2-AS1 |
| AC078883.1 |
| LENG8-AS1 |
| AC026412.3 |
| ACAP2-IT1 |
| GMDS-DT |
| MIR600HG |
| LINC02561 |
| AC144831.1 |
| AC073487.1 |
| AC005104.1 |
| AL670729.3 |
| AC021078.1 |
| AC245060.6 |
| AC008735.4 |
| MIR548XHG |
| AC055855.1 |
| AL133467.4 |
| AC008735.2 |
| AC015813.1 |
| AC116366.1 |
| AL137009.1 |
| AC116351.1 |
| MNX1-AS2 |
| AL354892.3 |
| AL139407.1 |
| AL354989.1 |
| DLEU1 |
| SBF2-AS1 |
| LINC01355 |
| LINC01224 |
| AC009061.2 |
| LINC00641 |
| TEX41 |
| AC026356.1 |
| NARF-IT1 |
| AL031670.1 |
| FAM111A-DT |
| AC025569.1 |
| SUGT1P4-STRA6LP |
| AC068756.1 |
| AC012360.3 |
| AL731533.2 |
| AC015849.3 |
| PTOV1-AS2 |
| KDM4A-AS1 |
| BACH1-IT1 |
| AF131215.6 |
| AC022031.2 |
